# Supplementary material for: Preparation and Performance of Ultra-Fine Polypropylene Antibacterial Fibers via Melt Electrospinning
Source: Polymers (Basel). 2020 Mar 6;12(3):606. doi: 10.3390/polym12030606 (PMC7182946; doi:10.3390/polym12030606)
Supplement: Supplementary file 1 [file polymers-12-00606-s001.pdf]

Supplementary Materials

Preparation and Performance of Ultra-Fine Polypropylene Antibacterial Fibers via Melt Electrospinning

Qiu-Sheng Li †, Hong-Wei He *,†, Zuo-Ze Fan, Ren-Hai Zhao, Fu-Xing Chen, Rong Zhou * and Xin Ning *

Industrial Research Institute of Nonwovens & Technical Textiles, College of Textiles & Clothing, Qingdao University, Qingdao 266071, China; 2017021401@qdu.edu.cn (Q.-S.L.); 2017021409@qdu.edu.cn (Z.-Z.F.); chinesezrh@126.com (R.-H.Z.); fuxing1991@gmail.com (F.-X.C.)

* Correspondence: hhwpost@163.com (H.-W.H.); rzhouqdu@126.com (R.Z.); xning@qdu.edu.cn (X.N.)

† These two authors contributed equally to this work.

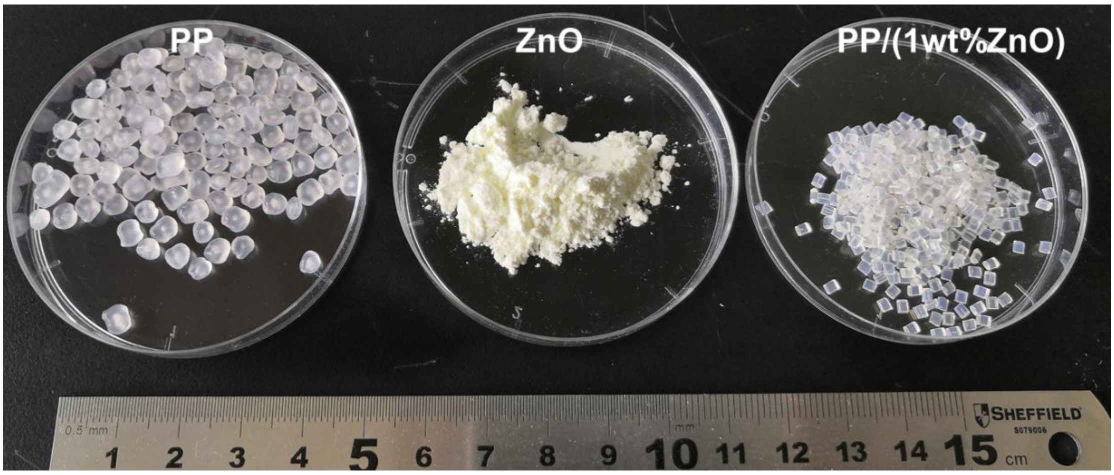

Figure S1. The digital camera photo of pristine PP, nano-ZnO and PP blended with 1wt% ZnO.

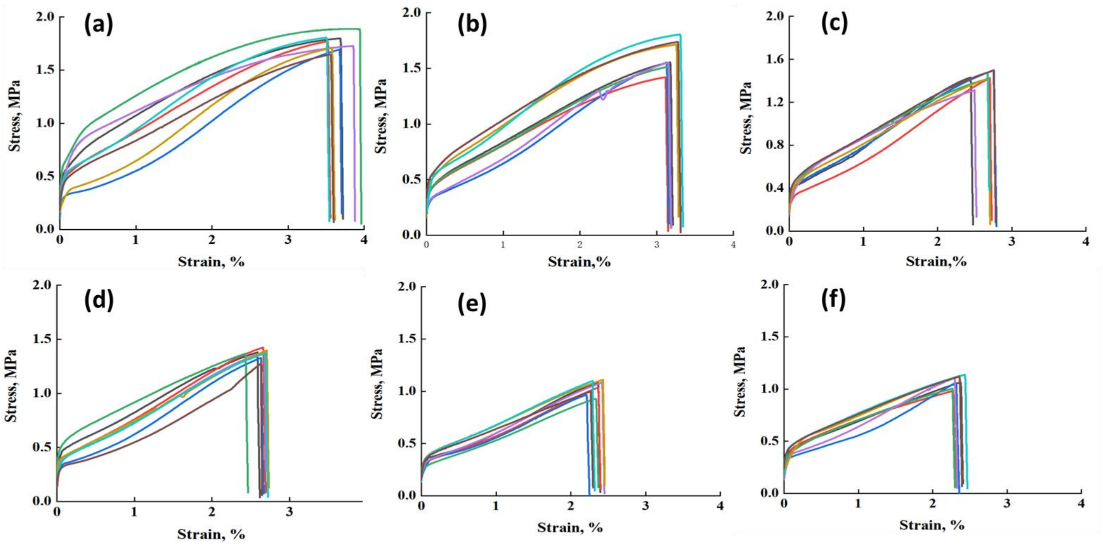

Figure S2. Stress-strain behaviors of e-spun PP fibers containing ZnO, (**a**):0 wt%, (**b**): 1 wt%, (**c**): 2 wt%, (**d**): 3 wt%, (**e**): 4 wt%, (**f**): 5 wt%, respectively. Every sample was tested 8 times.
